# Dietary inflammatory potential, systemic inflammation, and cognitive function in healthy adults in Saudi Arabia

**DOI:** 10.3389/fnut.2026.1853226

**Published:** 2026-07-17

**Authors:** Shoug Alashmali, Raneem Asiri

**Affiliations:** Department of Clinical Nutrition, Faculty of Applied Medical Sciences, King Abdulaziz University, Jeddah, Saudi Arabia

**Keywords:** cognitive performance, C-reactive protein, dietary inflammatory index, erythrocyte sedimentation rate, Montreal Cognitive Assessment, pro-inflammatory diet

## Abstract

**Objectives:**

To assess the relationship between the inflammatory potential of diets, measured using the energy-adjusted Dietary Inflammatory Index (E-DII), and serum high-sensitivity C-reactive protein (hs-CRP) level, erythrocyte sedimentation rate (ESR), and cognitive function, assessed using the Montreal Cognitive Assessment (MoCA).

**Methods:**

A subset of 117 adults (18–50 years old) from a larger sample completed a validated food frequency questionnaire (FFQ). Serum hs-CRP was measured using a high-sensitivity immunoturbidimetric method, and ESR was assessed using the Westergren method. Multiple linear regression analyses were conducted to evaluate associations between E-DII, inflammatory markers, and cognitive outcomes after adjusting for age, sex, education, income, body mass index, and smoking status. Sample sizes varied in the analyses due to different subsets of participants providing complete data.

**Results:**

Higher E-DII scores were positively associated with hs-CRP level (*β* = 0.445, 95% CI, 0.047–0.841; *p* = 0.029) but not with ESR. A higher E-DII was also associated with poorer performance in the MoCA language domain (*p* = 0.021), though no significant associations were found with total MoCA score. Additionally, hs-CRP was not significantly associated with total MoCA score.

**Conclusion:**

Pro-inflammatory dietary patterns are associated with higher systemic inflammation. However, no clear association was observed between dietary inflammatory potential/systemic inflammation, and cognitive function in this sample. Longitudinal and interventional studies with larger and more diverse populations, and incorporating additional inflammatory and cognitive biomarkers, are needed to further clarify these relationships.

## 1 Introduction

Globally, cognitive decline is a significant public health concern, with mild cognitive impairment (MCI) affecting approximately 23.7% of individuals above the age of 60 (1). In Saudi Arabia, the aging population has an increased prevalence of cognitive impairment. This was demonstrated in a community-based study conducted in Riyadh, where 45% of participants exhibited cognitive impairment, with 38.6% being diagnosed with MCI and 6.4% with dementia (2). These findings place Saudi Arabia within the upper range of global prevalence rates for cognitive impairment, underscoring the need for targeted interventions and public health strategies to address related risks.

Cognition is influenced by a complex interplay between various internal and external factors (3). Inflammation is one factor that appears to play a significant role in cognitive decline. Systemic inflammation, characterized by elevated levels of inflammatory biomarkers such as C-reactive protein (CRP), has been associated with increased cognitive decline over time (4). Inflammatory processes can promote neurodegeneration, such as that seen in Alzheimer’s disease (AD), and cognitive decline through various mechanisms, including the activation of pro-inflammatory microglia and astrocytes, tau hyperphosphorylation, and *β*-amyloid oligomerization (4).

Diet also has a potential effect on inflammation, and growing evidence suggests that inflammation, in turn, may influence cognitive function. The relationship between the inflammatory potential of the diet and cognition has been examined repeatedly in the literature, mostly in the context of older adults and people with dementia and AD. A longitudinal study involving 1,059 individuals with an average age of 73 years demonstrated that each additional unit of Dietary Inflammatory Index (DII) score was linked to a 21% higher risk of dementia incidence (5). In a separate study involving 7,085 women aged 65–79 years, researchers examined the connection between DII scores and MCI and dementia risk. Their findings revealed that diets with the highest DII scores were linked to an increased risk of MCI or dementia over a span of approximately 9.7 years (6). A meta-analysis that pooled data from 12 cross-sectional and prospective studies investigating the connection between DII and cognitive function found that individuals with higher DII scores were 1.34 times more likely to develop AD or MCI and 1.63 times more likely to experience global cognitive function impairment (7). In a separate meta-analysis of 9 cross-sectional and cohort studies, researchers observed that individuals with higher DII scores were 1.46 times more likely to develop cognitive impairment (8). Considering that these findings show a notable association between DII and cognitive impairment in different countries around the world, it is important to explore this relationship in the context of Saudi Arabia, especially given the current lack of studies on the topic in this region.

While cognitive decline is commonly acknowledged as a natural part of aging and most studies examining the connection between pro-inflammatory diets and cognitive function have primarily focused on the elderly population, it has been proposed that certain aspects of cognitive function may begin to decline in early adulthood (9). Moreover, other factors such as obesity and smoking have been reported to have a negative association with cognitive function in younger adults (10, 11), suggesting that the impact of dietary choices on cognitive health might be relevant to the younger population. The findings of this study have the potential to fill a critical research gap by focusing on a population primarily composed of healthy adults and whether diet can influence inflammation, thereby advancing the scientific understanding of the relationship between diet and cognition in this group. This study aims to investigate the relationship between the inflammatory potential of the diet and cognitive function in the healthy adult population living in Saudi Arabia.

## 2 Methods

### 2.1 Design and setting

This cross-sectional study included a subset of participants (*n* = 117) drawn from a larger, previously described sample (*n* = 256) (12). Participants were residents of Saudi Arabia and were recruited using convenience sampling. Recruitment was conducted using posters published on various social media platforms, including X (formerly Twitter), Instagram, WhatsApp, and Telegram, and posted at health events. Participants were incentivized to participate by offering them a free consultation with a dietitian with their laboratory results. Data collection ran from November 2024 to August 2025. Participants first completed an online form to assess their eligibility and collect sociodemographic, lifestyle, and dietary data, results of which were previously published (12). The form also provided them with details about the study’s objectives, a confidentiality statement, and a consent form, which they needed to review and acknowledge before proceeding. A subset of participants who were interested in proceeding further voluntarily provided their contact information at the end of the form and were later contacted for further data collection, administration of a cognitive assessment test, and blood sample extraction. This subset of participants comprises the sample for the present study. The study was conducted in accordance with the Declaration of Helsinki and approved by the Research Ethics Committee of the Unit of Biomedical Ethics at King Abdulaziz University (Reference No. 193-24).

### 2.2 Sample

This study included men and women aged 18–50 years residing in Saudi Arabia. Exclusion criteria included a history of attention-deficit/hyperactivity disorder, psychiatric disorders, AD, Down syndrome, multiple sclerosis, Parkinson’s disease, chronic diseases such as diabetes and cardiovascular diseases, alcohol or drug abuse, and the use of antipsychotic drugs or sedatives. Persons who did not speak Arabic were also excluded.

Sample size was calculated using G*Power Version 3.1.9.6 software (13). The required sample size was calculated based on multiple linear regression analysis for the relationship between energy-adjusted DII (E-DII) and Montreal Cognitive Assessment (MoCA) scores, as well as for the relationship between inflammatory marker levels, high-sensitivity C-reactive protein (hs-CRP) and erythrocyte sedimentation rate (ESR), and MoCA scores; controlling for six confounding factors (sex, age, education, income, smoking, and body mass index [BMI]) with a medium effect size (*f*^2^ = 0.15); *α* = 0.05; and 80% power. This resulted in a required sample of 103 participants. Our final sample of 117, therefore, provided sufficient power to detect these relationships. For the relationship between E-DII and hs-CRP level, we calculated the required sample size using an effect size of *f*^2^ = 0.27 (14), controlling for four confounding factors (sex, age, smoking, and BMI), *α* = 0.05, and 80% power, which resulted in a sample size of 54 participants. Our sample for this relationship consisted of 70 participants, which provided sufficient power. Since no published effect size estimates were available for the association between E-DII and ESR, no separate power calculation was conducted for ESR, which was, therefore, included as an exploratory outcome to examine potential associations with E-DII.

### 2.3 Recruitment and initial data collection

**Figure 1:**
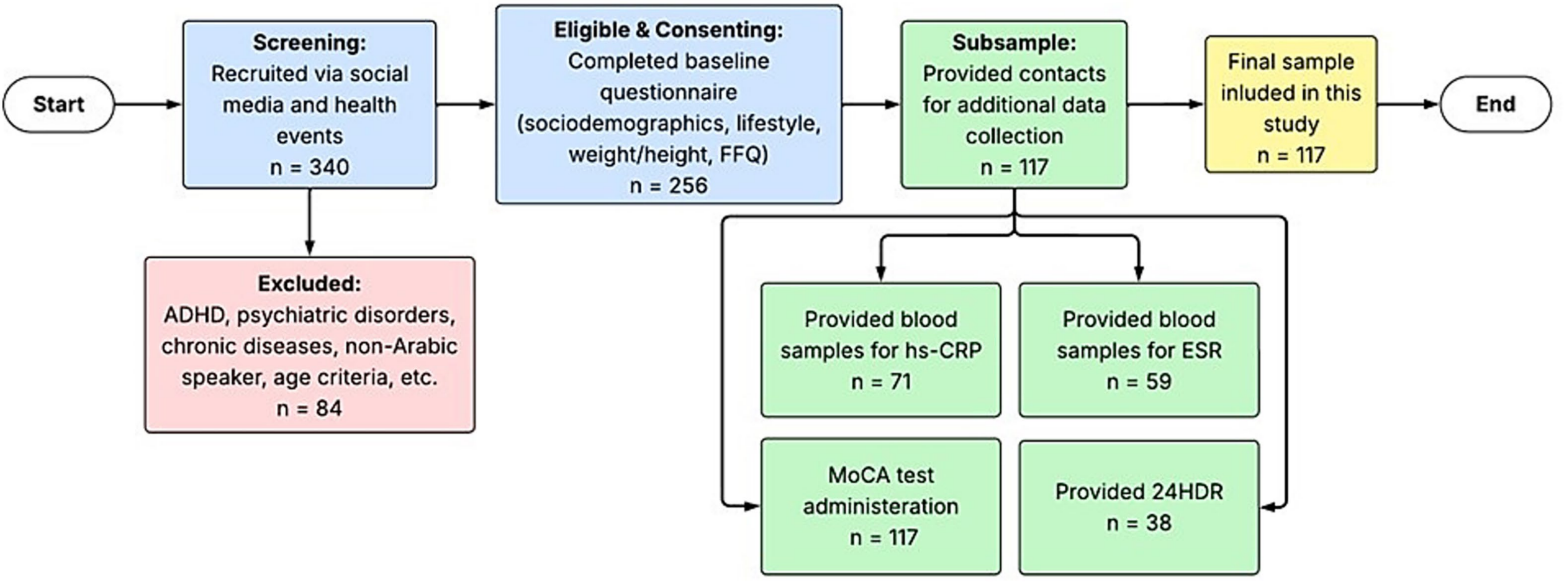
Flow chart of participant recruitment, screening, and inclusion. *n*, number of participants at each stage; FFQ, Food Frequency Questionnaire; 24hDR, 24-h Dietary Recall; hs-CRP, high-sensitivity C-reactive protein; ESR, erythrocyte sedimentation rate; MoCA, Montreal Cognitive Assessment test.

Participants who met the eligibility criteria and consented to participate provided demographic information, including age, sex, educational level, marital status, occupation, nationality, and income, as well as lifestyle factors such as smoking and supplement use, along with weight and height. Nutritional intake was evaluated through an electronic, validated food frequency questionnaire (FFQ). Participants who consented to further participation were contacted and invited to attend a laboratory visit. During the laboratory visit, additional data were collected, including a 24-h dietary recall (24hDR) (for a subset of participants), blood samples were drawn, and the participants were administered the MoCA cognitive test by trained personnel. The recruitment process, eligibility screening, exclusions, and final inclusion in the study are illustrated in Figure 1.

### 2.4 Dietary assessment

Dietary history was gathered using an electronic version of an FFQ developed and validated for use in Saudi Arabia (15). Used to calculate E-DII in local studies, the FFQ showed a significant positive association with serum hs-CRP (14, 16). The dietary assessment procedures described in detail elsewhere (12) were followed consistently in this study. Briefly, the FFQ assessed habitual dietary intake in the preceding 6 months and included 133 food items. Additional questions were included on selected items (e.g., tea, garlic, ginger, herbs, and spices) to enable a comprehensive calculation of the E-DII. Information on dietary supplement use, including type and amount consumed, was also collected. Standardized portion sizes specific to each food item were used to estimate individual intakes. Nutrient composition and overall dietary intake were calculated using Nutritics software (17), which incorporates a national food database for commonly consumed Saudi and Arabic foods (18). Intake data for flavonoids not listed in Nutritics were obtained from the U.S. Department of Agriculture’s flavonoid database (19). To reduce potential measurement error, a subsample of 15% of the original cohort completed a 24hDR gathered by a trained dietitian; this was used as a reference to calibrate FFQ-derived intakes using linear regression, as previously described (12). The calibrated FFQ values were used in subsequent analyses.

### 2.5 Dietary inflammatory index

The DII is a literature-derived tool, developed to assess the inflammatory potential of an individual’s diet, that has been widely used to examine associations between dietary patterns, inflammation, and health outcomes (20). The DII is based on scientific evidence linking specific dietary components to inflammatory biomarkers, and it incorporates a range of nutrients and bioactive compounds with established pro- or anti-inflammatory effects (20). In the present study, E-DII was calculated instead of DII to improve comparability across different energy intakes and provide better estimation of the diet’s inflammatory potential (21, 22). The E-DII was calculated using 42 dietary parameters available in the current dataset out of the 45 components of the original DII model; alcohol, eugenol, and isoflavones were not included due to lack of availability and cultural considerations relevant to the study population. Nutrient intake for different components was first energy-adjusted using the density method (per 1,000 kcal of total energy intake), then used to compute E-DII scores following the standardized DII scoring algorithm, as described previously (12). Positive E-DII values indicate a more pro-inflammatory diet, whereas negative values reflect a more anti-inflammatory dietary pattern. The DII has been shown to correlate with inflammatory biomarkers across diverse populations (23) and has previously demonstrated a positive association with serum hs-CRP levels in studies conducted in Saudi Arabia (14, 16).

### 2.6 Inflammatory biomarkers

Serum hs-CRP was analyzed as a biomarker of systemic inflammation. Blood samples were collected from participants by trained laboratory personnel at Shorooq Medical Laboratory in Jeddah, Saudi Arabia, following standard venipuncture procedures. Following centrifugation, serum samples were stored at −80 °C until the time of analysis. The concentration of hs-CRP (expressed in mg/L) was determined using a commercially available hs-CRP latex-enhanced immunoturbidimetric reagent kit compatible with the Thermo Scientific™ Indiko™ Plus Clinical Chemistry Analyzer (Thermo Fisher Scientific, Finland). Serum samples collected in serum separator tubes (SST) were analyzed according to the manufacturer. The assay quantitatively measures CRP based on the formation of antigen–antibody complexes. The resulting turbidity was measured photometrically at 340 nm. The assay had a detection limit of 1 mg/L, allowing for precise quantification of low-grade systemic inflammation. Serum ESR was measured using the Westergren method. Venous blood was collected into 3.2% sodium citrate tubes at a 4:1 blood-to-anticoagulant ratio and gently mixed immediately after collection. The samples were then allowed to stand vertically at room temperature for 30 min in accordance with the laboratory’s standard operating procedure. The erythrocyte sedimentation distance was measured and reported as mm/h. Quality control procedures were implemented according to laboratory standards, and all measurements were performed in duplicate to ensure the reliability and accuracy of results. hs-CRP measurements were available only for participants who attended the in-person laboratory visit, provided informed consent, and donated a blood sample for analysis. ESR was introduced as an exploratory biomarker after study initiation and was therefore measured only in participants recruited after its inclusion in the study protocol.

### 2.7 Cognitive function

The MoCA is a concise and validated screening tool designed for the evaluation of overall cognitive function. It is widely used across diverse clinical settings, playing an important role in the identification of cognitive impairment, including MCI and dementia, across different populations (24). It is sensitive enough to detect even subtle cognitive decline, particularly in the early stages of cognitive impairment (24). The MoCA provides a comprehensive evaluation, addressing various cognitive domains, such as visuospatial skills, attention, memory and delayed recall, language, abstraction, and orientation. The MoCA uses a point-based scoring system to assess these cognitive domains, with a maximum score of 30 points. The administration of the MoCA is straightforward, typically requiring 10–15 min for completion. For this study, a validated Arabic version of the MoCA (25) was administered by a trained professional during the on-site laboratory visits. The researcher completed the official MoCA training and certification required for its administration.

### 2.8 Statistical analysis

All statistical analyses were performed using IBM SPSS Statistics (26). Participant characteristics were presented using descriptive statistics. Continuous variables were presented as mean and standard deviation (SD) when normally distributed and as median and interquartile range (IQR) when non-normally distributed, while binary and categorical variables were expressed as counts and percentages.

Multiple linear regression analysis was used to assess the relationship between E-DII, cognitive function, and inflammatory biomarkers. Before the analysis, all key assumptions were assessed to ensure the validity of the models. Linearity between the independent and dependent variables was confirmed using scatterplots of residuals versus predicted values. The independence of residuals was tested with the Durbin-Watson statistic, which was within an acceptable range (1.5–2.5). Homoscedasticity, or equal variance of residuals, was checked by inspecting the spread of residuals across predicted values. Normality of residuals was examined using the Shapiro–Wilk test. No significant violations were detected. Statistical significance was set at *p* < 0.05 for all analyses.

To assess potential selection bias due to missing biomarker data, participants with and without hs-CRP availability were compared using chi-square tests/Fisher’s exact test for categorical variables and independent t-tests for continuous variables.

Sensitivity analyses were conducted to examine the robustness of the findings to extreme hs-CRP values. Specifically, analyses were repeated after excluding participants with hs-CRP concentrations greater than 15 mg/L. This threshold was selected based on previously published epidemiological studies applying similar cut-offs to reduce potential bias from acute infection or systemic inflammation (27).

#### 2.8.1 Associations between E-DII and inflammatory biomarkers

Linear regression analyses were conducted to examine the association between the E-DII and hs-CRP (the primary inflammatory outcome), adjusting for age, sex, smoking status, and BMI. A separate exploratory regression analysis was performed to assess the association between E-DII and ESR, using the same covariate adjustments.

#### 2.8.2 Associations between E-DII and cognitive performance

Linear regression analysis was used to evaluate the association between E-DII and global cognitive performance (assessed using the MoCA total score). Exploratory analyses were subsequently conducted to examine associations between E-DII and individual MoCA cognitive domains, including visuospatial ability, naming, attention, language, abstraction, delayed recall, memory index score (MIS), and orientation. All models were adjusted for age, sex, education, smoking status, BMI, and income.

#### 2.8.3 Associations between inflammatory biomarkers and cognitive performance

Linear regression analysis was used to assess the associations between hs-CRP and global cognitive performance, measured by the MoCA total score. Secondary exploratory analyses were conducted to examine associations between hs-CRP and individual MoCA domain scores (visuospatial ability, naming, attention, language, abstraction, delayed recall, memory index score (MIS), and orientation). In addition, exploratory analyses were performed to investigate associations between ESR and both MoCA total score and domain-specific scores. All models were adjusted for age, sex, education, smoking status, BMI, and income.

## 3 Results

### 3.1 Sample characteristics

Table 1 presents the sociodemographic and lifestyle characteristics of participants across the full cohort (*n* = 256) and the analytical subsamples included in the present study, including the MoCA subset (*n* = 117), hs-CRP subset (*n* = 70), and ESR subset (*n* = 59).

**Table 1:** Descriptive characteristics of the participants.

Variable	Full cohort (*n* = 256)	MoCA subset (*n* = 117)	hs-CRP subset (*n* = 70)	ESR subset (*n* = 59)
Sex, No. (%)				
Male	76 (29)	45 (38)	31 (44)	26 (44)
Female	180 (71)	72 (62)	39 (56)	33 (56)
Age group, No. (%), years				
18–29	116 (45)	57 (49)	28 (40)	21 (36)
30–39	79 (31)	30 (26)	18 (26)	17 (29)
40–50	61 (24)	30 (26)	24 (34)	21 (36)
Educational level, No. (%)				
High school	20 (8)	19 (16)	15 (21)	16 (27)
Bachelor	171 (67)	81 (69)	44 (63)	35 (59)
Postgraduate	65 (25)	17 (15)	11 (16)	8 (14)
Employment status, No. (%)				
Unemployed	70 (27)	34 (29)	22 (31)	21 (36)
Employed	149 (59)	67 (57)	37 (53)	29 (49)
Student	37 (14)	16 (14)	11 (16)	9 (15)
Income level, No. (%), SAR				
<4,000	95 (37)	42 (36)	29 (41)	27 (46)
4,001–10,000	98 (38)	56 (48)	27 (39)	25 (42)
>10,001	63 (25)	19 (16)	14 (20)	7 (12)
Region of residence, No. (%)				
Central	32 (13)	13 (11)	0 (0)	0 (0)
Western	186 (72)	97 (83)	64 (91)	57 (97)
Eastern	9 (4)	3 (3)	2 (3)	1 (2)
Southern	18 (7)	4 (3)	3 (4)	0 (0)
Northern	11 (4)	0 (0)	1 (1)	1 (2)
Marital status, No. (%)				
Single	133 (52)	63 (54)	36 (51)	29 (49)
Married	110 (43)	49 (42)	31 (44)	27 (46)
Separated	13 (5)	5 (4)	3 (4)	3 (5)
Nationality, No. (%)				
Saudi	210 (82)	83 (71)	49 (70)	34 (58)
Non-Saudi	46 (18)	34 (29)	21 (30)	25 (42)
Currently smokes tobacco, No. (%)				
Yes	29 (11)	22 (19)	17 (24)	12 (20)
No	227 (89)	95 (81)	53 (76)	47 (80)
BMI category, No. (%)				
Underweight	15 (6)	6 (5)	3 (4)	2 (3)
Normal	106 (41)	42 (36)	27 (39)	20 (34)
Overweight	80 (31)	44 (38)	25 (36)	24 (41)
Obese	55 (22)	25 (21)	15 (21)	13 (22)
E-DII score, mean (SD)	4.8 (1.3)	4.8 (0.8)	4.7 (0.8)	4.5 (0.7)
Biomarkers, median (IQR)				
hs-CRP level (mg/L)	–	–	1.6 (1.2, 2.9)	–
ESR (mm/h)	–	–	–	15 (5, 22)
MoCA score, median (IQR)				
Total score (out of 30)	–	26 (25, 28)	–	–
Visuospatial score (out of 5)	–	4 (3, 5)	–	–
Naming score (out of 3)	–	3 (3, 3)	–	–
Attention score (out of 6)	–	5 (4, 6)	–	–
Language score (out of 3)	–	2 (1, 3)	–	–
Abstraction score (out of 2)	–	2 (1, 2)	–	–
Delayed recall score (out of 5)	–	5 (5, 5)	–	–
MIS score (out of 15)	–	13 (11, 15)	–	–
Orientation score (out of 6)	–	6 (6, 6)	–	–

Subsamples are not mutually exclusive; participants may appear in more than one subgroup depending on available laboratory and cognitive data. hs-CRP, ESR, and MoCA scores are presented as median (IQR) due to skewed distributions. SD, standard deviation; IQR, interquartile range; E-DII, energy-adjusted Dietary Inflammatory Index; SAR, Saudi Riyal; BMI, body mass index; hs-CRP, high-sensitivity C-reactive protein; ESR, erythrocyte sedimentation rate; MoCA, Montreal Cognitive Assessment Test; MIS, memory index score.

Supplementary Table 1 presents the comparison of participants with available hs-CRP measurements (*n* = 70) and those without hs-CRP data (*n* = 186) to assess potential selection bias due to missing biomarker data. Participants were compared with respect to sociodemographic and lifestyle characteristics. There were no statistically significant differences between groups across any of the examined variables (all *p* > 0.05).

### 3.2 Associations between DII and sociodemographic characteristics

Associations between DII scores and sociodemographic characteristics have been described in detail (12) and are therefore not repeated here. Briefly, men had significantly higher E-DII scores, indicating more pro-inflammatory diets (*p* < 0.001). Additionally, subjects with a postgraduate educational level showed lower E-DII scores compared with participants with only a high school degree, reflecting more anti-inflammatory dietary patterns. However, this finding was not statistically significant (*p* = 0.06).

### 3.3 Associations between E-DII and inflammatory biomarkers

As shown in Table 2, hs-CRP levels were found to be significantly positively associated with E-DII scores. Specifically, for each one-unit increase in E-DII score, hs-CRP increased by 0.45 mg/L (*β* = 0.445, 95% CI, 0.047–0.841; *p* = 0.029) after adjusting for age, sex, smoking status, and BMI. However, ESR levels were not significantly associated with E-DII.

**Table 2:** Associations between E-DII score and hs-CRP and ESR levels.

Variable	*β*	95% CI	*p* value
Hs-CRP	0.445	0.047, 0.841	0.029^*^
ESR	0.007	−0.004, 0.021	0.216

Analysis based on *n* = 70 for hs-CRP and *n* = 59 for ESR. E-DII, energy-adjusted Dietary Inflammatory Index; hs-CRP, high-sensitivity C-reactive protein; ESR, erythrocyte sedimentation rate; *β*, unstandardized beta coefficient; CI, confidence interval. *p value < 0.05 indicates a statistically significant association.

### 3.4 Associations between E-DII and MoCA scores

As presented in Table 3, no significant associations were found between the total MoCA score and E-DII scores (*p* > 0.05), after adjusting for age, sex, education, income, smoking, and BMI. A significant negative association was observed in exploratory analyses between E-DII score and the language domain (*β* = −0.235, 95% CI: −0.434 to −0.035, *p* = 0.021), adjusted for the same covariates. No significant associations were observed for the remaining exploratory cognitive domain scores.

**Table 3:** Associations between E-DII score and MoCA cognitive domain and total scores.

MoCA score	*β*	95% CI	*p* value
Total score	−0.021	−0.658, 0.606	0.934
Domain scores			
Visuospatial	−0.086	−0.320, 0.148	0.468
Naming	0.017	−0.058, 0.092	0.651
Attention	0.253	−0.083, 0.591	0.139
Language	−0.235	−0.434, −0.035	0.021^*^
Abstraction	−0.033	−0.169, 0.103	0.633
Delayed recall	0.075	−0.026, 0.176	0.145
MIS	0.128	−0.445, 0.700	0.659
Orientation	−0.035	−0.135, 0.065	0.490

Analysis based on *n* = 117. E-DII, Energy-adjusted dietary inflammatory index; MoCA, Montreal Cognitive Assessment test; *β*, unstandardized beta coefficient; CI, confidence interval; MIS, Memory index score. *p value < 0.05 indicates a statistically significant association.

**Figure 2:**
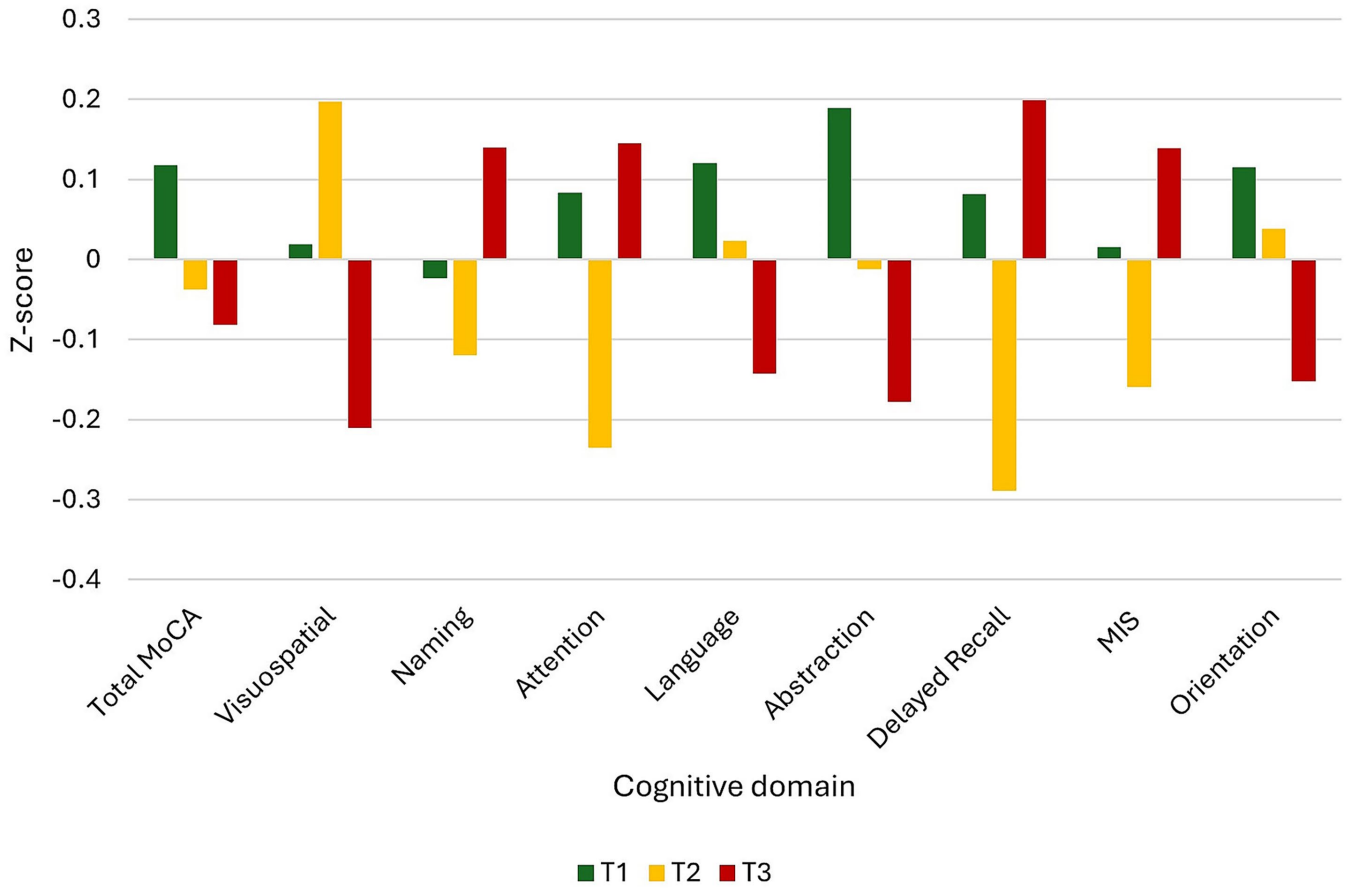
Mean MoCA total and domain z-scores across tertiles of the E-DII. E-DII ranged between 2.2 and 6.9 scores. T1–T3 represent tertiles of E-DII scores from least to most pro-inflammatory. T1 includes scores from 2.2 to 4.4 (least inflammatory diet), T2 from 4.5 to 5.0 (moderate inflammatory diet), and T3 from 5.0 to 6.9 (most inflammatory diet). MoCA, Montreal Cognitive Assessment test; E-DII, energy-adjusted dietary inflammatory index; MIS, Memory Index Score.

Mean MoCA total and domain z scores across E-DII tertiles are shown in Figure 2. The graph shows a consistent trend of gradual decrease across E-DII tertiles in the total MoCA scores as well as scores for the language, abstraction, and orientation domains. In contrast, the remaining domains did not exhibit a clear or consistent pattern across tertiles.

### 3.5 Associations between inflammatory biomarkers and MoCA scores

As shown in Table 4, no significant associations were observed between hs-CRP level and MoCA total or exploratory analysis of the domain scores after controlling for age, sex, education, smoking status, BMI, and income (*p* > 0.05). The findings of the sensitivity analysis excluding participants with extreme hs-CRP concentrations were consistent with the main analyses, with no meaningful changes in the direction or magnitude of the observed associations (Supplementary Table 2).

**Table 4:** Associations between hs-CRP level and MoCA total and cognitive domain scores.

MoCA score	*β*	95% CI	*p* value
Total score	0.053	−0.027, 0.133	0.188
Domain scores			
Visuospatial	−0.018	−0.048, 0.011	0.221
Naming	0.002	−0.001, 0.012	0.661
Attention	0.026	−0.021, 0.072	0.281
Language	0.020	−0.007, 0.046	0.140
Abstraction	0.014	−0.004, 0.032	0.127
Delayed recall	0.013	−0.005, 0.031	0.135
MIS	0.049	−0.035, 0.133	0.248
Orientation	−0.004	−0.013, 0.005	0.395

Analysis based on *n* = 70. hs-CRP, high-sensitivity C-reactive protein; MoCA, Montreal Cognitive Assessment test; *β*, unstandardized beta coefficient; CI, confidence interval; MIS, memory index score.

For ESR, the exploratory analysis of delayed recall domain showed a statistically significant positive association with ESR level (*β* = 0.015, 95% CI, 0.003–0.026; *p* = 0.015) as presented in Table 5. The remaining domain and total MoCA scores showed no significant association with ESR after controlling for age, sex, education, smoking status, BMI, and income (*p* > 0.05).

**Table 5:** Associations between ESR level and MoCA total and cognitive domain scores.

MoCA score	*β*	95% CI	*p* value
Total score	0.021	−0.034, 0.075	0.454
Domain scores			
Visuospatial	−0.002	−0.020, 0.016	0.795
Naming	−0.003	−0.010, 0.003	0.268
Attention	0.008	−0.025, 0.040	0.749
Language	0.002	−0.013, 0.018	0.140
Abstraction	0.004	−0.007, 0.016	0.471
Delayed recall	0.015	0.003, 0.026	0.015^*^
MIS	0.043	−0.010, 0.096	0.106
Orientation	−0.003	−0.007, 0.002	0.248

Analysis based on *n* = 59. ESR, erythrocyte sedimentation rate; MoCA, Montreal Cognitive Assessment test; *β*, unstandardized beta coefficient; CI, confidence interval; MIS, memory index score. *p value < 0.05 indicates a statistically significant association.

## 4 Discussion

### 4.1 Main findings

This study examined associations between dietary inflammatory potential, as assessed by the E-DII, inflammatory biomarkers, and cognitive scores among adults residing in Saudi Arabia. A significant positive association was observed between E-DII and hs-CRP levels, suggesting that more pro-inflammatory diets were associated with higher systemic inflammation. No significant associations were observed between E-DII and total MoCA score, or between hs-CRP and cognitive function.

### 4.2 Interpretation of findings in relation to previous studies

Consistent with previous research, higher E-DII scores were significantly associated with higher inflammatory marker levels such as hs-CRP, interleukin-6 (IL-6), and tumor necrosis factor (TNF) *α* receptor 2 (16, 27). For example, in a sample of 2,567 postmenopausal women, higher DII scores were significantly associated with higher levels of IL-6, TNF-α receptor 2, and hs-CRP (27). In another study, E-DII was significantly associated with higher levels of hs-CRP in men (23). Similarly, a study including over 20,000 adults showed that increased DII scores were associated with higher composite scores for low-grade inflammation (including CRP, platelets, leukocyte count, and granulocyte-to-lymphocyte ratio) (28). This indicates that more pro-inflammatory diets are linked to increased systemic inflammation. Additionally, these findings support the usefulness of the DII as a valid tool for capturing the inflammatory potential of a diet. In contrast, the lack of a significant association between E-DII and ESR level may be explained by fundamental differences between these biomarkers. While hs-CRP is a sensitive acute-phase reactant that responds rapidly to dietary and metabolic influences (29), ESR is a less specific marker influenced by factors such as age, sex, anemia, and plasma protein composition (30). Moreover, ESR was included as an exploratory biomarker in the present study, and the absence of prior effect size estimates may have limited the ability to detect a statistically significant association (47). Taken together, these findings suggest that hs-CRP may be a more responsive biomarker than ESR for capturing diet-related inflammatory changes in relatively healthy adult populations.

The observed association between dietary inflammatory potential and hs-CRP aligns with mechanistic evidence suggesting that diet can modulate systemic inflammation through multiple interconnected biological pathways. Diets with higher inflammatory potential, characterized by lower intake of fruits, vegetables, whole grains, and higher consumption of red meat, saturated fatty acids, simple sugars, fried foods, and ultra-processed products have been shown to promote systemic low-grade inflammation and oxidative stress through increased production of pro-inflammatory cytokines, including TNF-*α*, IL-6, and IL-1β, which activate downstream signaling pathways such as NF-κB, thereby sustaining inflammatory responses (31). Oxidative stress represents a closely related mechanism through which dietary patterns may influence systemic and brain health. Excess production of reactive oxygen species (ROS) leads to oxidative damage of lipids, proteins, and DNA, and further amplifies inflammatory signaling via activation of pathways such as NF-κB (32, 33). This bidirectional interaction between inflammation and oxidative stress creates a self-perpetuating cycle that may contribute to chronic low-grade inflammatory states. In addition to immune and oxidative pathways, diet may influence neurobiological outcomes through effects on the gut microbiota. Dietary patterns high in fat and low in fiber can induce gut dysbiosis, characterized by reduced microbial diversity and increased abundance of pro-inflammatory bacteria (34). Dysbiosis may increase intestinal permeability, facilitating translocation of lipopolysaccharide (LPS) into the circulation and activation of Toll-like receptor-mediated inflammatory pathways, thereby contributing to systemic inflammation (31). Conversely, dietary fiber promotes the production of short-chain fatty acids (SCFAs), including butyrate, which exert anti-inflammatory effects through inhibition of NF-κB signaling and enhancement of gut barrier integrity (35). These metabolites also reduce endotoxin translocation and support immune regulation (35). Beyond systemic effects, emerging evidence suggests that gut-derived metabolites and inflammatory mediators may influence brain function through the gut-brain axis. Increased intestinal permeability and dysbiosis-associated metabolites may enter systemic circulation and cross the blood–brain barrier, where they can contribute to neuroinflammatory processes and negatively affect neurogenesis and neuronal function (36). This pathway provides a biologically plausible link between dietary inflammatory potential, systemic inflammation, and brain health outcomes. Overall, these mechanisms support a diet-inflammation-oxidative stress-gut-brain axis framework, providing biological plausibility for the associations observed in the present study. This is consistent with recent evidence highlighting the role of dietary patterns in modulating inflammatory and oxidative pathways relevant to cognitive health (31, 37).

The present study found no significant association between E-DII scores and global cognitive performance in a relatively young and healthy adult population. This finding differs from a substantial body of epidemiological evidence suggesting that higher DII scores are associated with poorer cognitive outcomes, particularly in older adults and clinical populations at higher risk of cognitive decline (5–8). For example, a recent meta-analysis of observational studies reported that higher DII scores were associated with increased odds of cognitive impairment, including mild cognitive impairment and Alzheimer’s disease, as well as poorer global cognitive function in some analyses, although substantial heterogeneity across studies was noted (7). Similarly, another meta-analysis found that pro-inflammatory diets were associated with an increased risk of AD, MCI, and global cognitive impairment, supporting a potential link between dietary inflammation and cognitive health, while also highlighting variability in effect estimates across populations and study designs (7). However, systematic reviews indicate that findings are not fully consistent across all cognitive domains and study types, with differences likely related to variations in cognitive assessment methods and study design, including cross-sectional versus longitudinal approaches (8). The absence of an association in the present study may reflect the relatively young age and generally healthy participants, as well as limited variability in cognitive performance in non-clinical populations. It is also possible that longer exposure to dietary inflammatory patterns or age-related vulnerability is required before effects on global cognitive function become detectable. The MoCA was used in the present study as a global screening measure of cognitive function due to its practicality and relatively higher sensitivity compared with other brief screening tools such as the Mini-Mental State Examination. However, it is important to acknowledge that MoCA is primarily designed for the detection of cognitive impairment rather than the assessment of subtle inter-individual differences in cognitive performance within relatively young and cognitively healthy populations (24). In such samples, overall scores and several cognitive domains may demonstrate ceiling effects and restricted variability (38), which can limit the ability to detect small but potentially meaningful differences in cognitive function. This methodological consideration should be taken into account when interpreting the absence of associations with total MoCA score and the exploratory nature of domain-level findings in the present study. In exploratory, unadjusted analyses, a statistically significant association at the nominal level was observed between E-DII and the MoCA language domain, while no significant associations were found for total MoCA score or other cognitive domains. Some previous studies have reported associations between dietary inflammatory potential and specific cognitive domains, although findings have been heterogeneous. For instance, a large cohort study of 3,080 participants, over a 13-year follow-up, found that pro-inflammatory diets assessed at midlife were linked to poorer verbal memory but not executive functioning (39). Similarly, a study of approximately 2,700 adults aged 60 years and older found that higher DII scores were associated with poorer performance on the Digit Symbol Substitution Test and Animal Fluency, both reflecting processing speed and verbal fluency; while associations with other cognitive tests, including word learning and delayed recall, were not significant (40). The observed association with the language domain may reflect variability in the sensitivity of different cognitive domains to dietary-related inflammatory processes; however, this interpretation remains speculative given the exploratory nature of the analysis. For example, longitudinal studies in older adults have reported that lower verbal fluency scores may precede the development of MCI or Alzheimer’s disease (41, 42). Although these studies were conducted in older populations and are not directly comparable to the present sample, they suggest that language-related measures may be sensitive to early cognitive changes in general.

The absence of a significant association between hs-CRP level and MoCA scores in our study may be due to several factors. Most studies reporting significant effects involved larger samples, longitudinal follow-up, or older populations (43, 44). Furthermore, physical activity, sleep quality, stress, or other inflammatory markers may have influenced the observed associations (45, 46). A meta-analysis reported a significant association between elevated CRP levels and cognitive decline; however, the effect size was small, indicating a weak relationship and that the link between CRP and cognitive deterioration in individuals without dementia appears to be marginal (44). This may help explain the lack of a significant association in our relatively young and healthy sample. Furthermore, it is possible that systemic inflammation alone does not fully capture the complex biological pathways linking diet and cognition. In exploratory, unadjusted analyses, a nominally significant association was observed between ESR and delayed recall; however, the effect size was very small, and the result should be interpreted cautiously given the exploratory nature of the analysis and the lack of adjustment for multiple comparisons.

### 4.3 Limitations

This study has several limitations that should be considered when interpreting its findings. The cross-sectional design limits the study’s ability to infer causal relationships between systemic inflammation, dietary inflammatory potential, and cognitive function. Based on the chosen design, measurements of hs-CRP and E-DII reflect only a single time point and may not capture long-term patterns that could influence cognition. Furthermore, the use of convenience sampling may have introduced selection bias, as participation was voluntary and restricted to individuals who responded to the online survey. Consequently, the study sample may differ from the general population in unmeasured characteristics, which may limit the generalizability of the findings. Moreover, the relatively small sample size for the analysis of MoCA scores in relation to hs-CRP and ESR may have limited the statistical power to detect subtle associations. Moreover, hs-CRP showed a right-skewed distribution, which is common in population-based studies; however, sensitivity analyses produced consistent results, suggesting that this did not significantly affect the findings. While efforts were made to adjust for key demographic and lifestyle factors, residual confounding cannot be excluded. Specifically, variables such as physical activity, sleep quality, and psychological stress were not available in the present dataset and may have influenced both systemic inflammation and cognitive outcomes. Additional inflammatory markers were also not assessed, which may have further contributed to unmeasured variability in the observed associations. Furthermore, the study population was healthy, with a female majority, which may limit the generalizability of the findings to other populations, such as those with chronic diseases or older age groups at higher risk for cognitive decline. Additionally, self-reported dietary data used to calculate the E-DII are subject to recall bias or measurement error, potentially affecting the accuracy of the dietary inflammatory estimates. Despite these limitations, the study provides useful insights into the relationship between systemic inflammation, dietary inflammatory potential, and cognitive function, highlighting areas for future research.

### 4.4 Implications

The findings of this study have several important implications for research and public health. The observed positive association between dietary inflammatory potential and systemic inflammation, as reflected by hs-CRP level, supports the role of diet as a modifiable determinant of low-grade systemic inflammation. Promoting diets rich in fruits, vegetables, whole grains, nuts, legumes, and healthy fatty acids may serve as an accessible strategy to improve health among adults in Saudi Arabia. In contrast, no significant associations were observed between E-DII and global cognitive performance, nor between hs-CRP and cognitive scores. This may suggest that, in younger and relatively healthy adults, cognitive function is not yet sensitive to variation in dietary inflammatory exposure or circulating inflammatory markers. It is also possible that longer exposure duration or greater biological vulnerability, such as aging or clinical risk status, may be required for associations with cognition to become detectable in this population. Future studies should consider integrating multiple biomarkers, such as IL-1, IL-6, and TNF-*α*. as well as neuroimaging and longitudinal follow-up, to better understand the underlying mechanisms. Finally, these findings highlight the need for larger, longitudinal studies in diverse populations in Saudi Arabia to confirm the observed associations and explore potential mediating factors such as physical activity, sleep, and stress.

## 5 Conclusion

This study investigated the relationship between dietary inflammatory potential, systemic inflammation, and cognitive performance among adults living in Saudi Arabia. The findings revealed that higher E-DII scores, reflecting more pro-inflammatory dietary patterns, were significantly associated with elevated hs-CRP levels, supporting the link between diet and systemic inflammation. These findings suggest that interventions aiming to reduce dietary inflammation could have measurable effects on biomarkers like hs-CRP. Furthermore, no significant associations were found between E-DII and overall cognitive performance, or between hs-CRP and cognitive performance. Overall, these results highlight that while dietary inflammatory potential is reflected in systemic inflammatory status, its relationship with cognitive function may be less apparent in younger and generally healthy adult populations. These findings also underscore the importance of promoting anti-inflammatory dietary patterns, rich in fruits, vegetables, whole grains, and healthy fatty acids, as an approach to reduce systemic inflammation. Future research should aim to confirm these findings in larger, more diverse populations and across different age groups. Incorporating additional biomarkers of inflammation, oxidative stress, and neurodegeneration, as well as objective measures of dietary intake, would enhance the accuracy and depth of future investigations. Finally, exploring potential mediators, such as gut microbiota, metabolic health, and genetic predisposition, could provide valuable insights into the biological mechanisms linking diet, inflammation, and cognitive function.

## Data Availability

The datasets presented in this article are not readily available because the data that support the findings of this study are available on request from the corresponding author. Requests to access the datasets should be directed to Shoug Alashmali, smsaleh1@kau.edu.sa.
