# Supplementary material for: Dietary inflammatory potential, systemic inflammation, and cognitive function in healthy adults in Saudi Arabia
Source: Front Nutr. 2026 Jul 17;13:1853226. doi: 10.3389/fnut.2026.1853226 (PMC13423657; doi:10.3389/fnut.2026.1853226)
Supplement: Supplementary file 1 [file Supplementary_File_1.docx]

**Supplementary**

**Table 1. Comparison of participant characteristics according to availability of hs-CRP data**

| **Variable** | **hs-CRP Available (n=70)** | **hs-CRP Missing (n=186)** | ***p value*** |
| --- | --- | --- | --- |
| **Sex, No. (%)** |  |  | 0.767 |
| Male | 31 (44) | 140 (75) |  |
| Female | 39 (56) | 46 (25) |  |
| **Age Group, No. (%), years** |  |  | 0.941 |
| 18-29 | 28 (40) | 88 (47) |  |
| 30-39 | 18 (26) | 60 (32) |  |
| 40-50 | 24 (34) | 38 (20) |  |
| **Educational Level, No. (%)** |  |  | 0.336 |
| High school | 15 (21) | 6 (3) |  |
| Bachelor | 44 (63) | 126 (68) |  |
| Postgraduate | 11 (16) | 54 (29) |  |
| **Employment Status, No. (%)** |  |  | 0.516 |
| Unemployed | 22 (31) | 46 (25) |  |
| Employed | 37 (53) | 113 (61) |  |
| Student | 11 (16) | 27 (15) |  |
| **Income Level, No. (%), SAR** |  |  | 0.858 |
| <4000 | 29 (41) | 68 (37) |  |
| 4001-10,000 | 27 (39) | 69 (37) |  |
| >10,001 | 14 (20) | 49 (26) |  |
| **Region of Residence, No. (%)** |  |  | 0.341 |
| Central | 0 (0) | 33 (18) |  |
| Western | 64 (91) | 121 (65) |  |
| Eastern | 2 (3) | 7 (4) |  |
| Southern | 3 (4) | 15 (8) |  |
| Northern | 1 (1) | 10 (5) |  |
| **Marital Status, No. (%)** |  |  | 0.502 |
| Single | 36 (51) | 97 (52) |  |
| Married | 31 (44) | 79 (43) |  |
| Separated | 3 (4) | 10 (5) |  |
| **Nationality, No. (%)** |  |  | 0.822 |
| Saudi | 49 (70) | 163 (88) |  |
| Non-Saudi | 21 (30) | 23 (12) |  |
| **Currently Smokes Tobacco, No. (%)** |  |  | 0.568 |
| Yes | 17 (24) | 13 (7) |  |
| No | 53 (76) | 173 (93) |  |
| **BMI Category, No. (%)** |  |  | 0.800 |
| Underweight | 3 (4) | 14 (8) |  |
| Normal | 27 (39) | 81 (44) |  |
| Overweight | 25 (36) | 53 (29) |  |
| Obese | 15 (21) | 38 (20) |  |
| E-DII score, mean (SD) | 4.7 (0.8) | 4.9 (0.8) | 0.347 |

**Note**. Differences between groups were assessed using chi-square tests for categorical variables, except for region of residence, where Fisher’s exact test was used due to small expected cell counts. Continuous variables were compared using independent samples t-tests. Statistical significance was set at p < 0.05. SD = standard deviation; E-DII = energy-adjusted Dietary Inflammatory Index; SAR = Saudi Riyal; BMI = body mass index.

**Table 2. Sensitivity analysis excluding hs-CRP > 15 mg/L**

| **Model** | **β** | **95% CI** | ***p value*** |
| --- | --- | --- | --- |
| hs-CRP → E-DII | 0.450 | 0.06, 0.84 | 0.023* |
| hs-CRP → total MoCA score | 0.084 | -0.18, 0.35 | 0.532 |

**Note**. E-DII = Energy-adjusted dietary inﬂammatory index; hs-CRP = high-sensitivity C-reactive protein; MoCA = Montreal Cognitive Assessment test; β = unstandardized beta coefficient; CI = confidence interval.
